# Potential Antibacterial Activity of Carvacrol-Loaded Poly(DL-lactide-*co*-glycolide) (PLGA) Nanoparticles against Microbial Biofilm

**DOI:** 10.3390/ijms12085039

**Published:** 2011-08-08

**Authors:** Antonio Iannitelli, Rossella Grande, Antonio Di Stefano, Mara Di Giulio, Piera Sozio, Lucinda Janete Bessa, Sara Laserra, Cecilia Paolini, Feliciano Protasi, Luigina Cellini

**Affiliations:** 1 Department of Drug Sciences, Faculty of Pharmacy; University G. d’Annunzio, Via dei Vestini 31, Chieti 66100, Italy; E-Mails: aiannitelli@unich.it (A.I.); r.grande@unich.it (R.G.); m.digiulio@unich.it (M.G.D.); p.sozio@unich.it (P.S.); ljbesa@unich.it (L.J.B.); s.laserra@unich.it (S.L.); l.cellini@unich.it (L.C.); 2 CeSI-Center for Research on Ageing & DNI-Department of Neuroscience and Imaging, University G. d’Annunzio, Chieti-Pescara, Via dei Vestini 31, Chieti 66100, Italy; E-Mails: cpaolini@unich.it (C.P.); fprotasi@unich.it (F.P.)

**Keywords:** carvacrol, biofilm, PLGA nanoparticles

## Abstract

The ability to form biofilms contributes significantly to the pathogenesis of many microbial infections, including a variety of ocular diseases often associated with the biofilm formation on foreign materials. Carvacrol (Car.) is an important component of essential oils and recently has attracted much attention pursuant to its ability to promote microbial biofilm disruption. In the present study Car. has been encapsulated in poly(dl-lactide-*co*-glycolide (PLGA) nanocapsules in order to obtain a suitable drug delivery system that could represent a starting point for developing new therapeutic strategies against biofilm-associated infections, such as improving the drug effect by associating an antimicrobial agent with a biofilm viscoelasticity modifier.

## 1. Introduction

Biofilms are an organized community of bacterial cells embedded within a hydrated matrix of self-produced extracellular polymeric substances (EPS), which preferentially develop at solid/liquid interfaces. The typical components of EPS, polysaccharides, proteins and DNA, provide structural stability and protection to the biofilm while the presence of water channels within the community structure, resembling a primitive circulatory system, ensures nutrients and waste products diffusion gradients [1,2]. The clinical relevance of these microbial communities is related to their wide ocurrence in the developed world together with their ability to cause relapsing infections characterized by chronic inflammation, tissue damage and the significant difficulties in their eradication [3,4]. In fact, a wide number of diseases, of which bronchopneumonia in cystic fibrosis patients [5], chronic rhino-sinusitis [6], and endocarditis [7] represent a short list of examples, result directly from or are associated with biofilm growth of bacteria. In addition, owing to their ability to colonize the outer and/or inner surface of medical devices such as catheters [8] and shunts [9], respirators, contact lenses [10], heart valves, stents, and orthopedic prostheses [11,12], biofilms are important in that they are responsible for medical device-related infections.

The ability to form biofilms significantly alters important microbial properties: sessile cells show less susceptibility to antibiotics and the minimal inhibitory concentration (MIC) and minimal bactericidal concentration (MBC) of antibiotics to biofilm-growing bacteria may be up to 10-fold higher compared with planktonic bacteria. Moreover, phagocytic host defense mechanism seems incapable of removing the biofilm cells protected by the EPS matrix [13–15]. The extraordinary tolerance displayed by cells in the biofilm towards antimicrobials and the difficulties encountered in eradication of biofilm-related infections have been ascribed to a combination of factors, such as high levels of metabolically active and growing cells at the biofilm surface and low or no growth in the centre giving rise to a subpopulation of persister cells [16,17], high mutation frequency of biofilm-growing bacteria allowing the emergence of antibiotic resistance [18], and the presence of the EPS matrix that, being rich in antibiotic-inactivating enzymes and efflux pumps, plays the role of a diffusion barrier [19]. There is, hence, widespread recognition that alternative strategies or more effective agents exhibiting activity against biofilm-growing microorganisms are required in order to face the unmet medical needs for effective novel antibacterial agents. In this regard, Car. [2-methyl-5-(1-methylethyl)phenol], the main component of several essential oils, attracted considerable interest for its wide spectrum of antimicrobial activity [20]. Importantly, it has been reported that Car. can inhibit the growth of preformed biofilms and to interfere with biofilm formation [21–24]. More recently, Nostro and coworkers reported the ability of Car. (1% v/v) to significantly reduce *S. aureus* and *S. epidermidis* biofilm biomass and cultivable cell numbers altering the dense matrix of the biofilm and the cell morphology [25].

A great contribution to the improvement of the therapies for biofilm-related diseases is expected with the application of nanotechnology to the pharmaceutical sciences. Owing to their tunable size, increased suspendability, surface tailorability that allows interactions with biological systems at the molecular level [26], nanoparticulate devices could diffuse into the mucus environment surrounding the biofilm colonies and there exploit the local and controlled release of the anti biofilm ingredient increasing its residence time and effectiveness [27–29]. Notably, Cheow and coworkers reported the preparation of levofloxacin-loaded poly(dl-lactide-*co*-glycolide) (PLGA) and poly(caprolactone) (PLC) nanoparticles and showed that, to be effective against *E. coli* biofilm cells, the formulation must ensure a biphasic extended release profile with an initial fast release providing high antibiotic concentrations for the biofilm eradication, followed by slower antibiotic extended release able to minimize biofilm growth and infection exacerbation [30]. Recently, Keawchaoon described the preparation of carvacrol-loaded chitosan nanoparticles with antimicrobial activity against planktonic *S. aureus, B. cereus* and *E. coli* cells [31]. The present research, in order to combine the anti biofilm activity of Car. with the excellent delivery features of nanotechnology devices and the optimal biocompatibility of PLGA polymers [32], focuses on the fabrication of carvacrol-loaded PLGA nanocapsules. The nanodevices have been fabricated by the solvent displacement method, characterized by dynamic light scattering (DLS), zeta potential measurements, and transmission electron microscopy (T.E.M.), and in terms of encapsulation efficiency (EE%), drug loading capacity (DL%) and *in vitro* release profile. Finally, the effects of Car. and carvacrol-loaded nanocapsules (Car.-Nps) on the viscoelastic features of *S. epidermidis* ATCC 35984 biofilms grown under dynamic conditions have been investigated by rheological measurements. A cone/plate rheometer was used to measure the viscoelastic properties of the staphylococcal biofilms produced. This technique affords the precise monitoring of shear stress and strain acting on the sample [33], and provides the opportunity to elucidate, at a macroscopic level, the ability of Car. to destabilize the architecture of the dense matrix in which biofilm cells are embedded.

## 2. Results and Discussion

### 2.1. Preparation and Characterization of Car.-Nps

**Figure 1.**
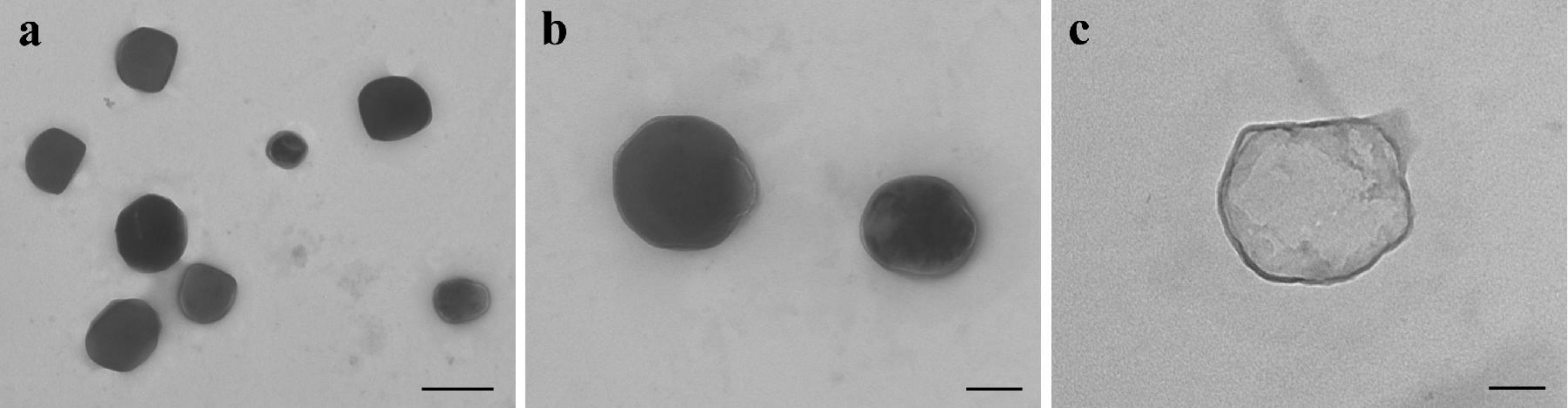
Electron micrographs showing groups of two or more nanoparticles at different magnifications (1a and 1b). In (1c), high magnification (×140 k) of a Car.-Np. Scale bars: (**a**) 0.5 μm; (**b**) 0.2 μm; (**c**) 50 nm.

In the present work Car.-Nps were conveniently prepared in a good yield (80%) by the solvent displacement method. Owing to its lipophilic nature Car. was encapsulated with satisfying EE% and DL% (Table 1). More precisely, this method allows the formation of spherical nanocapsules where the oily core composed of Car., considered to be a volatile molecule (vapor pressure is 0.0232 mmHg at 25 °C), is entrapped and retained in a thin dense wall formed by PLGA polymer and phosphatidylcholine. Examination by T.E.M. confirmed the presence of nanocapsules characterized by electron-dense walls (Figure 1a,b). The individual particles exhibited an approximately spherical shape and a quite variable diameter. The nanocapsules tend to cluster on the grid, and showed a compact/thick layer, formed by PLGA and phosphatidylcholine (Figure 1a,b), surrounding the less electrondense and thin core of Car. reservoir (Figure 1c).

The formulation was able to undergo the freeze-drying process yielding spherical Car.-Nps characterized by a relatively narrow size distribution and a mean hydrodynamic diameter (209.8 ± 7.2 nm, Table 1), that could allow the diffusion of the particles through the mucus layers present on the surface of anatomical sites more frequently subjects of biofilm infections, such as lung airways, sinuses, eyes, and gastrointestinal tract. In fact, having a diameter in the range of 200 nm is one of the requisite for nanoparticulate devices to reach the surface of mucosal districts covered by this critical barrier [27,28].

### 2.2. *In Vitro* Drug Release

**Figure 2.**
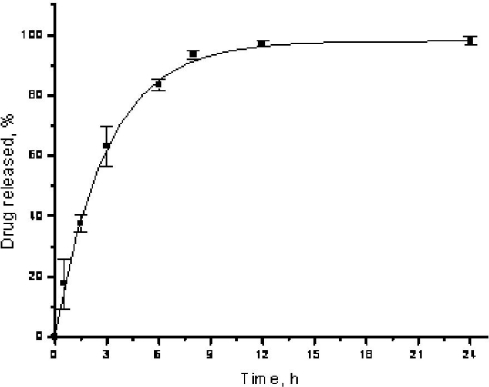
Cumulative Car. release from PLGA nanocapsules in PBS pH 7.4 containing 0.5% Tween 80 at 37 ± 0.5 °C (*n* = 3).

The in vitro release profile of Car.-Nps was investigated in phosphate buffer solution, pH 7.4 containing 0.5% Tween 80 (PBST) and at 37 ± 0.5 °C. The total drug amount inside the nanocapsules was kept below 10% of the drug solubility limit in PBS in order to ensure sink conditions. The in vitro release behavior of Car.-Nps presented as the cumulative percentage release is shown in Figure 2. Drug release occurred with an initial “burst” release followed by a slower release due to the concentration gradient. Car.-Nps show a 60% release after 3 h, and approach to completeness after 24 h with approximately 95% of Car. released. The considerable initial fast release of Car. from the PLGA nanocapsules might be ascribed to the physicochemical properties of the encapsulated drug. In fact, Car., having a log P_o/w_ of 3.64 will partition into the lipophilic matrix of the thin polymeric membrane surrounding the inner compartment of the carriers and, owing to its water solubility of 0.83 mg/mL, could diffuse with high rate toward the aqueous outer phase [34].

Importantly, Car. is reported to exert its antimicrobial and biofilm-destabilization effects within the first 3 h after cell exposition [25], thus, Car.-Nps, being characterized by a “burst” effect in their drug release kinetic, could ensure adequate Car. concentration soon after their administration in order to exert its antimicrobial effect.

### 2.3. Biofilm Growth and Rheological Tests

**Figure 3.**
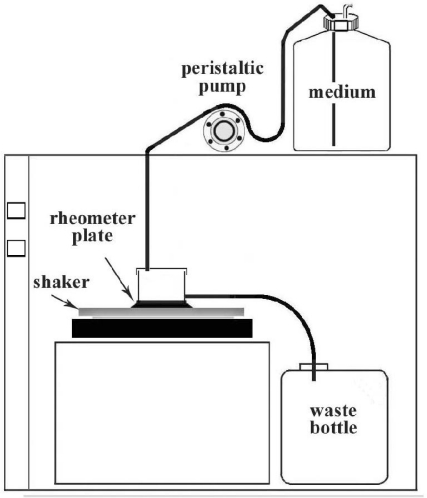
Flow system apparatus.

Rheometry is a well-established technique for measuring properties of viscoelastic samples, and it has the advantage that shear stresses and strain acting on the sample are precisely monitored. The rheological properties measured are shown in Table 2. All the samples tested were grown directly on the rheometer measuring plate in order to preserve their structure, and under a continuous flow rate to better reproduce the *in vivo* conditions (See experimental section and Figure 3).

Samples were divided into biofilms not exposed to Car. (Bio), biofilms treated with Car. itself (Bio + Car.), and biofilms treated with Car.-Nps (Bio + Car.-Nps), and showed a linear viscoelastic range (LVR). The region was characterized by stress-strain linear proportionality and by constant values of the elastic (G′) and the viscous (G″) moduli, starting from 1.0 Pa up to 10.0 Pa and in the frequency range 0.1–10 Hz. Biofilms from 48 h growth in dynamic conditions (Figure 3, see experimental section: *Biofilm preparation*) displayed, from a macroscopic point of view, a compact structure which correlates with the viscoelastic behavior observed during the rheological tests. These findings are in agreement with the results reported in the literature for biofilms of other bacterial species [35–43] where the creep and recovery curves of bacterial biofilms showed the typical time-dependent behavior of viscoelastic materials having a gel-like structure. It is observed that both biofilms treated either with Car. or Car.-Nps showed a statistically significant reduction in the values of the steady-flow viscosity when compared with biofilm alone; this finding allows us to speculate a possible role of Car. in triggering structural modifications on the EPS matrix of bacterial biofilms. Similarly, the equilibrium compliance, *J_e0_*, a measure of the elasticity of viscoelastic materials at short times, [44] was reduced by the presence of Car., but the data are significant only for Car.-Nps.

Rheological measurements in the dynamic mode at a constant shear stress indicated a considerable elasticity and mechanical stability for the investigated biofilms and confirmed the gel-like behavior with the G′ dominating the G″ by one order of magnitude and exhibiting little frequency dependence over the range of angular frequencies tested. Biofilms exposed to Car. were still characterized by gel-like structure and behavior, but showed lower values of G′ and G″ with Car.-Nps significantly affecting the magnitude of both the moduli. These new mechanical properties of the biofilm treated with Car.-Nps reflect alterations in the architecture of the biofilm and correspond to a fluidification of the EPS matrix that could potentially enable or enhance the penetration of antimicrobial agents into the deep core of the biofilm, where the persister cells are located. These cells are characterized by an absent/reduced metabolism that makes them tolerant of the action of the majority of the antimicrobial agents [45]; thus Car., which exerts its activity on structural and functional properties of cell membranes independently of the cell status, [25] may play a crucial role against the pool of persister cells that in most cases represent the main obstacle to an effective biofilm eradication.

Oscillation stress sweeps in the shear stress range of 1.0–400 Pa at the fixed frequency of 1.0 Hz were performed to identify the yield point (τ_y_) of the biofilms. Although the procedure to identify the τ_y_ provides results influenced by the experimental condition used (operative frequencies influence the results), the τ_y_ values might provide a means to quantify the strength with which biofilms adhere to solid surfaces at the solid-liquid interfaces. All the tested biofilms showed high values of τ_y_ indicating a strong ability to adhere to the stainless steel surfaces. Car. treatment seems unable to induce a statistically significant reduction of the strength with which preformed biofilms adhere to the measuring plates. Car. effectiveness is probably more evident at the early stage of biofilm formation, when the adherence capacity of planktonic cells represents a major step in the sessile colony formation [46]. In our experiments, instead, *S. epidermidis* ATCC 35984 biofilms were exposed to the action of Car. at the late stage of their development, when the adhesion process to solid surfaces is complete.

Biofilms produced in different laboratories are greatly heterogeneous [47], but a commonality of the elastic relaxation times (λ, the time scale separating solid and fluid behavior) of different biofilms can be observed. Indeed the biofilms’ elastic relaxation times found in our measurements are in accordance with the mean value of 18 min reported by Shaw *et al.* for a wide sample of biofilms (Table 2) [39].

## 3. Experimental Section

### 3.1. Materials

PLGA (d,l-lactide 50: glycolide 50, inherent viscosity within 0.15–0.25 dL/g) with free carboxyl end-groups was purchased from Lactel Absorbable Polymers (Pelham, USA). Soy lecithin (phosphatidylcholine-enriched fraction, Epikuron 200), was a kind gift of Cargill Inc (Italy). Carvacrol, acetone, Pluronic^®^ F-68, membrane dialysis (molecular weight cut-off 12,400), Tween^®^ 80, and HPLC grade methanol were purchased from Sigma-Aldrich (Milan, Italy).

### 3.2. Preparation of Nanocapsules

Nanocapsules containing carvacrol were prepared using the solvent displacement process described by Fessi *et al.* [48] Briefly, PLGA (55 mg), Epikuron 200 (65 mg), Car. (244 mg) were dissolved in acetone (12.5 mL), and the resulting solution poured, under moderate magnetic stirring, into 25 mL of an aqueous solution of Pluronic^®^ F68 5% (w/v). Following 10 min of stirring, the volume of nanocapsules dispersion was concentrated to 10 mL under reduced pressure. Separation of non-encapsulated compounds, as well as removal of the exceeding surfactant was performed by dialysis technique introducing the suspension containing the nanocapsules (10 mL) in a dialysis tube hermetically sealed and dialyzing at 37 °C against 3 × 1000 mL of deionized water for 24 h under magnetic stirring. Next, the samples collected were freeze-dried during 24 h to obtain a fine powder of nanoparticles. All samples were prepared and purified in triplicate. The recovery of nanocapsules was calculated as the ratio of the amount of recovered freeze-dried nanoparticles to the total amount of polymer and drug added.

### 3.3. Characterization of Car.-Nps

The size, size distribution and Zeta potential of reconstituted nanocapsules-freeze-dried were measured using a Brookhaven (90PLUS BI-MAS) digital correlator equipped with a He-Ne (*è Ne o N_2_*) laser operating at wavelength of 660.0 nm and at the scattering angle of 90°. All measurements were performed at 25 °C and performed at least in triplicate.

### 3.4. Preparation and Analysis of Car.-Nps by T.E.M

Sample dispersions were dropped onto a copper grid pre-coated with carbon, and dried under reduced pressure prior to T.E.M. observation. Samples were then examined with a Morgagni Series 268D electron microscope (FEI Company, Brno, Czech Republic), equipped with Megaview III digital camera at an accelerating voltage of 100 kV.

### 3.5. Determination of Car. Loading

The Car. loading was determined quantifying the drug extracted from the nanocapsules as reported elsewhere [49]. Briefly, 10 mg of dried nanocapsules was dissolved in 5 mL of acetone, stirred for 10 min and sonicated for 20 min. Next, the dispersion was filtered (membrane filter pore size 0.22 μm) and the resulting clear solution was analyzed by HPLC for Car. content using a Waters 1525 binary pump equipped with a Waters 2996 photodiode array detector and a Waters Symmetry C18 RP column (4.6 × 150 mm, 5 μm). A mixture of water and methanol (30:70) was used as eluent (flow rate 1.0 mL/min, detection wavelength 274.5 nm). Calibration was done with standard dissolved in acetone. The experiment was performed in triplicate. DL% and EE% of Car. were calculated from Equations (1) and (2), respectively:

(1) $$\text{DL}\%=\frac{\text{mass of encapsulated carvacrol}}{\text{mass of produced nanocapsules}}\times 100$$

(2) $$\text{EE}\%=\frac{\text{mass of encapsulated carvacrol}}{\text{mass of added carvacrol}}\times 100$$

### 3.6. *In Vitro* Drug Release

The dialysis bag diffusion technique was used to study the in vitro drug release of Car.-Nps [50]. Freeze-dried nanocapsules were re-suspended in PBS and aliquots were placed in dialysis tubes (cellulose membrane, molecular weight cut-off 12,000), hermetically sealed, and immersed into the dissolution medium [PBS, pH 7.4 containing Tween^®^ 80 0.5% (PBST)]. The total drug amount inside the nanocapsules was kept below 10% of the drug solubility limit in order to ensure sink conditions. The entire system was kept at 37 ± 0.5 °C with continuous magnetic stirring at 40 rpm. The dialysis tubes were collected from the dissolution medium at predetermined time intervals and assayed for Car. content as reported above. Briefly, 0.5 mL of suspension containing the nanocapsules were dissolved in 4.5 mL of acetone, stirred for 10 min and sonicated for 20 min. Next, the dispersion was filtered (membrane filter pore size 0.22 μm) and the resulting clear solution was analyzed by HPLC for Car. content. The cumulative percentage of Car. released from the nanocapsules at a certain time interval was calculated by Equation (3):

(3) Cumulative%carvacrol released=100−(%carvacrol remaining)

### 3.7. Biofilm Preparation

The reference strain *Staphylococcus epidermidis* ATCC 35984 (biofilm producer) [51] was the microorganism used for the experiments. For the biofilm preparation, a bacterial colony was cultured overnight at 37 °C in Tryptic Soy Broth (TSB) (CA; Oxoid Ltd, Italy), then diluted 1:10 (v/v) in TSB plus 0.25% of glucose (TSBG) for 2 h at 37 °C in an orbital shaker at 160 r.p.m.. The refreshed broth culture was adjusted to OD_600_ 0.25 (*ca.* 3 × 10^8^ CFU mL^−1^) in TSBG and inoculated on the rheometer plate for the biofilm formation. The biofilm was grown directly on the rheometer plate surface, in order to preserve its structure intact in the flow system apparatus (Figure 3). After a bacterial attachment period of 4 h on the rheometer plate in static condition at 37 °C, an orbital shaker was activated at 40 r.p.m., and a peristaltic pump was used to provide a continuous flow of sterile 1/10^th^ strength of TSB supplemented with 0.25% glucose, at the rate of 60 mL h^−1^ [41], for 48 h at 37 °C. After 48 h of biofilm growth, the flow was stopped and the mature biofilm was washed with sterile PBS and treated with 1/10^th^ strength of TSBG (10 mL) containing Car. or Car.-Nps (Car. final concentration 0.25% v/v) for 6 h, in an orbital shaker at 40 r.p.m.. This concentration represents the dose of 8 MIC, as previously determined [24]. The control was treated with 1/10^th^ strength of TSBG, in the same conditions.

### 3.8. Rheological Tests

The viscoelastic properties of bacterial biofilms were evaluated using a Haake M.A.R.S. II Thermo Scientific modular rheometer equipped with a cone and plate sensor system as reported previously [51]. Briefly, the low non-rotating element that received the sample was a stainless steel plate (Din No.1.4841). To prevent dehydration of the biofilm, the sample sandwiched between the cone, and the plate was surrounded by 10 mL of sterile PBS at 37 °C. In order to minimize the ‘squeezing G’ of water out of the macropores and channels of the biofilm, the rate by which the upper sensor reached the measurement position was controlled so that the normal force acting on the sample did not exceed the value of 0.1 N. The samples were allowed to undergo an ‘adjustment’ period of 5 min after loading [40]. All tests were carried out at the controlled temperature of 37 ± 0.3 °C, with the aid of a Haake Phoenix (Thermo Electron Corporation) thermo-controller system. The RheoWin software 3.61 (Thermo Fisher Scientific) was used for data evaluation. Creep and recovery tests were performed, applying a constant shear stress (*τ*) for 300 s followed by a 300 s recovery period (*τ* = 0); the resulting shear strain (*γ*) was measured over time (*t*). These tests were performed at several different *τ* (allowing the samples to undergo an adjustment period of 300 s between each test), in order to identify the linear viscoelastic range (LVR) of the samples, where γ was directly proportional to *τ*. Dynamic tests were carried out in order to estimate the elastic (*G*′) and the viscous (*G*″) moduli of the biofilms, and for the determination of their LVR. A series of oscillation frequency sweep tests at shear stress in the range 1.0–10.0 Pa, across a frequency range from 0.1 to 10 Hz (previously identified as the frequency range satisfying the linear viscoelasticity for all the evaluated biofilms), was performed for each sample, and the complex shear viscosity (η*), the elastic and the viscous moduli were measured as a function of frequency. Oscillation stress sweeps were carried out in the shear stress range 1.0–400 Pa at the fixed frequency of 1.0 Hz to evaluate the yield points of the biofilms with τ_y_ value being identified as the lowest stress value to which G′ become dependent on stress amplitude. The measurements were carried out in the LVR, as otherwise the results would be dependent on the experimental details and not unique to the material [44].

### 3.9. Data analysis and Statistics

Obtained data were expressed as the mean ± standard error of the mean (SEM) and compared by one-way ANOVA followed by Tukey’s test. Differences were considered significant at *P* < 0.05.

## 4. Conclusions

The aim of this study was to design polymeric nanocapsules loaded with the poorly soluble antimicrobial agent carvacrol. Assembling the results presented, it can be concluded that carvacrol encapsulation into PLGA nanocapsules was accomplished with good drug loading and encapsulation efficiency. The obtained particles had a spherical shape with a hydrodynamic diameter of about 210 nm, which could enable their diffusion through mucus layers present on the surface of anatomical sites. Electron microscopy revealed that the nanocapsules were characterized by an oily core of carvacrol surrounded by a compact thin polymeric wall. Rheological tests revealed the ability of carvacrol loaded nanocapsules to alter the properties of preformed staphylococcal biofilms: treatment with carvacrol loaded nanocapsules produced a considerable reduction in the elasticity and mechanical stability of preformed biofilms that could enable the penetration of antimicrobial agents into the deep core of bacterial biofilms. In conclusion, these new nanodevices merit further investigation as the delivery system of antimicrobial agents due to the unique possibility of co-encapsulate, in addition to carvacrol, a further antibacterial agent. In fact, the oily core formed by carvacrol, would act as a drug reservoir of the additional antibacterial agent opportunely encapsulated during the formulation procedure.

## Figures and Tables

**Table 1.** Characteristics of the Car.-Nps formulation. Data are expressed as mean ± SD (*n* = 3).

**Particle size (nm)**	**Polydispersity**	**Zeta potential (mV)**	**DL%**	**EE%**
209.8 ± 7.2	0.260 ± 0.013	−18.99 ± 3.01	21	26

**Table 2.** Rheological characterization of the *S. epidermidis* ATCC 35984 biofilms. Data are expressed as mean ± SD (*n* = 5).

**Sample**	**LVR (Pa)**	***η*_0_ (Pa s)**	***J*_e0_ (Pa^−1^)**	***λ* (min)**	***G′* (Pa)^1^**	**G″ (Pa)^1^**	**τ_y_ (Pa)^2^**
Bio	1–10	(1.1 ± 0.2) × 10^6^	(1.1 ± 0.3) × 10 ^−3^	21.9 ± 6.2	(1.3 ± 0.3) × 10^3^	(1.3 ± 0.5) × 10^2^	(2.3 ± 0.3) × 10^2^
Bio + Car.	1–10	(6.9 ± 0.6) × 10^5^^a^	(2.5 ± 0.5) × 10 ^−3^	21.6 ± 4.4	(9.3 ± 1.5) × 10^2^	(0.9 ± 0.1) × 10^2^	(1.5 ± 0.5) × 10^2^
Bio + Car.-Np	1–10	(5.5 ± 1.7) × 10^5^^a^	(7.9 ± 2.8) × 10 ^−3^^a^^,^^b^	25.5 ± 7.3	(6.1 ± 1.2) × 10^2^^a^	(0.6 ± 0.1) × 10^2^^a^	(1.3 ± 0.6) × 10^2^

1. shear stress (τ) 1.0 Pa satisfying the linear viscoelasticity: angular frequency (*ω*) varied from 0.1 to 10 Hz.

2. τ range 1.0–400 Pa, ω = 1.0 Hz.

a *p* < 0.05 compared with Bio.

b *p* < 0.05 compared to Car.

## References

[1] Lawrence JR, Korber DR, Hoyle BD, Costerton JW, Caldwell DE (1991). Optical sectioning of microbial biofilms. J. Bacteriol.

[2] De Beer D, Stoodley P, Roe F, Lewandowski Z (1994). Effects of biofilm structures on oxygen distribution and mass transport. Biotechnol. Bioeng.

[3] Costerton W, Veeh R, Shirtliff M, Pasmore M, Post C, Ehrlich G (2003). The application of biofilm science to the study and control of chronic bacterial infections. J. Clin. Invest.

[4] Mulcahy LR, Burns JL, Lory S, Lewis K (2010). Emergence of pseudomonas aeruginosa strains producing high levels of persister cells in patients with cystic fibrosis. J. Bacteriol.

[5] Bjarnsholt T, Jensen PØ, Fiandaca MJ, Pedersen J, Hansen CR, Andersen CB, Pressler T, Givskov M, Høiby N (2009). Pseudomonas aeruginosa Biofilms in the Respiratory Tract of Cystic Fibrosis Patients. Pediatr. Pulmonol.

[6] Sanderson AR, Leid JG, Hunsaker D (2006). Bacterial biofilms on the sinus mucosa of human subjects with chronic rhinosinusitis. Laryngoscope.

[7] Catalanotti P, Lanza M, Del Prete A, Lucido M, Catania MR, Gallè F, Boggia D, Perfetto B, Rossano F (2005). Slime-producing Staphylococcus epidermidis and S. aureus in acute bacterial conjunctivitis in soft contact lens wearers. New Microbiol.

[8] Passerini L, Lam K, Costerton JW, King EG (1992). Biofilms on indwelling vascular catheters. Crit. Care Med.

[9] Pople IK, Bayston R, Hayward RD (1992). Infection of cerebrospinal fluid shunts in infants: a study of etiological factors. J. Neurosurg.

[10] Okajima Y, Kobayakawa S, Tsuji A, Tochikubo T (2006). Biofilm formation by Staphylococcus epidermidis on intraocular lens material. Invest. Ophthalmol. Vis. Sci.

[11] Gristina AG, Oga M, Webb LX, Hobgood CD (1985). Adherent bacterial colonization in the pathogenesis of osteomyelitis. Science.

[12] Del Pozo JL, Patel R (2009). Infection associated with prosthetic joints. N. Engl. J. Med.

[13] Davies D (2003). Understanding biofilm resistance to antibacterial agents. Nat. Rev. Drug Discov.

[14] Moskowitz SM, Foster JM, Emerson J, Burns JL (2004). Clinically feasible biofilm susceptibility assay for isolates of Pseudomonas aeruginosa from patients with cystic fibrosis. J. Clin. Microbiol.

[15] Høiby N, Bjarnsholt T, Givskov M, Molin S, Ciofu O (2010). Antibiotic resistance of bacterial biofilms. Int. J. Antimicrob. Agents.

[16] Keren I, Kaldalu N, Spoering A, Wang Y, Lewis K (2004). Persister cells and tolerance to antimicrobials. FEMS Microbiol. Lett.

[17] Balaban NQ, Merrin J, Chait R, Kowalik L, Leibler S (2004). Bacterial persistence as a phenotypic switch. Science.

[18] Driffield K, Miller K, Bostock M, O’Neill AJ, Chopra I (2008). Increased mutability of Pseudomonas aeruginosa in biofilms. J. Antimicrob. Chemother.

[19] Bagge N, Hentzer M, Andersen JB, Ciofu O, Givskov M, Hoiby N (2004). Dynamics and spatial distribution of β-lactamase expression in Pseudomonas aeruginosa biofilms. Antimicrob. Agents Chemother.

[20] Lambert RJW, Skandamis PN, Coote PJ, Nychas GJE (2001). A study of the minimum inhibitory concentration and mode of action of oregano essential oil, thymol and carvacrol. J. Appl. Microbiol.

[24] Nostro A, Sudano Roccaro A, Bisignano G, Marino A, Cannatelli MA, Pizzimenti FC, Cioni PL, Procopio F, Blanco AR (2007). Effects of oregano, carvacrol and thymol on Staphylococcus aureus and Staphylococcus epidermidis biofilms. J. Med. Microbiol.

[25] Nostro A, Marino A, Blanco AR, Cellini L, Di Giulio M, Pizzimenti F, Sudano Roccaro A, Bisognano G (2009). In vitro activity of carvacrol against staphylococcal preformed biofilm by liquid and vapour contact. J. Med. Microbiol.

[26] Mansour HM, Sohn M, Al-Ghananeem A, DeLuca PP (2010). Materials for pharmaceutical dosage forms: Molecular pharmaceutics and controlled release drug delivery aspects. Int. J. Mol. Sci.

[27] Suk JS, Lai SK, Wang YY, Ensign LM, Zeitlin PL, Boyle MP, Hanes J (2009). The penetration of fresh undiluted sputum expectorated by cystic fibrosis patients by non-adhesive polymer nanoparticles. Biomaterials.

[28] Tang BC, Dawson M, Lai SK, Wang YY, Suk JS, Yang M, Zeitlin P, Boyle MP, Fu J, Hanes J (2009). Biodegradable polymer nanoparticles that rapidly penetrate the human mucus barrier. Proc. Natl. Acad. Sci. USA.

[29] Meers P, Neville M, Malinin V, Scotto AW, Sardaryan G, Kurumunda R, Mackinson GJ, Fisher S, Perkins WR (2008). Biofilm penetration, triggered release and *in vivo* activity of inhaled liposomal amikacin in chronic Pseudomonas aeruginosa lung infections. J. Antimicrob. Chemoth.

[30] Cheow WS, Chang MW, Hadinoto K (2010). Antibacterial efficacy of inhalable levofloxacin-loaded polymeric nanoparticles against E. coli biofilm cells: The effect of antibiotic release profile. Pharm. Res.

[31] Keawchaoon L, Yoksan R (2011). Preparation, characterization and in vitro release study of carvacrol-loaded chitosan nanoparticles. Colloids Surf. B.

[32] Anderson J, Shive M (1997). Biodegradation and biocompatibility of PLA and PLGA microspheres. Adv. Drug Deliv. Rev.

[33] Towler BW, Rupp CJ, Cunningham AB, Stoodley P (2003). Viscoelastic properties of a mixed culture biofilm from rheometer creep analysis. Biofouling.

[34] Griffin SG, Wyllie SG, Markham JL, Leach D (1999). The role of structure and molecular properties of terpenoids in determining their antimicrobial activity. Flavour Fragr. J.

[35] Stoodley P, Lewandowski Z, Boyle JD, Lappin-Scott HM (1999). Structural deformation of bacterial biofilms caused by short-term fluctuations in fluid shear: an in situ investigation of biofilm rheology. Biotechnol. Bioeng.

[36] Stoodley P, Cargo R, Rupp CJ, Wilson S, Klapper I (2002). Biofilm material properties as related to shear- induced deformation and detachment phenomena. J. Ind. Microbiol. Biotechnol.

[37] Korstgens V, Flemming HC, Wingender J, Borchard W (2001). Uniaxial compression measurement device for investigation of the mechanical stability of biofilms. J. Microbiol. Methods.

[38] Klapper I, Rupp CJ, Cargo B, Purvedorj R, Stoodley P (2002). Viscoelastic fluid description of bacterial biofilm material properties. Biotechnol. Bioeng.

[39] Shaw T, Winston M, Rupp CJ, Klapper I, Stoodley P (2004). Commonality of elastic relaxation times in biofilms. Phys. Rev. Lett.

[40] Vinogradov AM, Winston M, Rupp CJ, Stoodley P (2004). Rheology of biofilms formed from the dental plaque pathogen Streptococcus mutans. Biofilms.

[41] Rupp CJ, Fux CA, Stoodley P (2005). Viscoelasticity of Staphylococcus aureus biofilms in response to fluid shear allows resistance to detachment and facilitates rolling migration. Appl. Environ. Microbiol.

[42] Towler BW, Cunningham A, Stoodley P, McKittrick L (2007). Amodel of fluid–biofilm interaction using a Burger material law. Biotechnol. Bioeng.

[43] Yarwood JM, Paquette KM, Tikh IB, Volper EM, Greenberg EP (2007). Generation of virulence factor variants in Staphylococcus aureus biofilms. J. Bacteriol.

[44] Barnes HA, Hutton JF, Walters K, Barnes HA, Hutton JF, Walters K (1989). Linear viscoelasticity. An Introduction to Rheology.

[45] Lewis K (2010). Persister cells. Annu. Rev. Microbiol.

[46] Dalleau S, Cateau E, Bergès T, Berjeaud JM, Imbert C (2008). *In vitro* activity of terpenes against Candida biofilms. Int. J. Antimicrob. Agents.

[47] Houari A, Picard J, Habarou H, Galas L, Vaudry H, Heim V, Di Martino P (2008). Rheology of biofilms formed at the surface of NF membranes in a drinking water production unit. Biofouling.

[48] Fessi H, Puisieux F, Devissaguet JP, Ammoury N, Benita S (1989). Nanocapsule formation by interfacial polymer depositino following solvent displacement. Int. J. Pharm.

[49] Matsumoto J, Nakada Y, Sakurai K, Nakamura T, Takahashi Y (1999). Preparation of nanoparticles consisted of poly(l-lactide)–poly(ethylene glycol)–poly(L-lactide) and their evaluation *in vitro*. Int. J. Pharm.

[50] Verger MLL, Fluckiger L, Kim YI, Hoffman M, Maincent P (1998). Preparation and characterization of nanoparticles containing an antihypertensive agent. Eur. J. Pharm. Biopharm.

[51] Di Stefano A, D’Aurizio E, Trubiani O, Grande R, Di Campli E, Di Giulio M, Di Bartolomeo S, Sozio P, Iannitelli A, Nostro A, Cellini L (2009). Viscoelastic properties of Staphylococcus aureus and Staphylococcus epidermidis mono-microbial biofilms. Microb. Biotechnol.

